# Do Pioneer Cells Exist?

**DOI:** 10.1371/journal.pone.0085488

**Published:** 2014-01-21

**Authors:** Matthew J. Simpson, Parvathi Haridas, D. L. Sean McElwain

**Affiliations:** 1 School of Mathematical Sciences, Queensland University of Technology, Brisbane, Queensland, Australia; 2 Institute of Health and Biomedical Innovation (IHBI), Queensland University of Technology, Brisbane, Queensland, Australia; Dalhousie University, Canada

## Abstract

Most mathematical models of collective cell spreading make the standard assumption that the cell diffusivity and cell proliferation rate are constants that do not vary across the cell population. Here we present a combined experimental and mathematical modeling study which aims to investigate how differences in the cell diffusivity and cell proliferation rate amongst a population of cells can impact the collective behavior of the population. We present data from a three-dimensional transwell migration assay that suggests that the cell diffusivity of some groups of cells within the population can be as much as three times higher than the cell diffusivity of other groups of cells within the population. Using this information, we explore the consequences of explicitly representing this variability in a mathematical model of a scratch assay where we treat the total population of cells as two, possibly distinct, subpopulations. Our results show that when we make the standard assumption that all cells within the population behave identically we observe the formation of moving fronts of cells where both subpopulations are well-mixed and indistinguishable. In contrast, when we consider the same system where the two subpopulations are distinct, we observe a very different outcome where the spreading population becomes spatially organized with the more motile subpopulation dominating at the leading edge while the less motile subpopulation is practically absent from the leading edge. These modeling predictions are consistent with previous experimental observations and suggest that standard mathematical approaches, where we treat the cell diffusivity and cell proliferation rate as constants, might not be appropriate.

## Introduction

Collective cell spreading plays an important role in development [1], repair [2]–[5] and disease [6]. One way of improving our understanding of the mechanisms that influence collective cell spreading is to develop and implement a mathematical model that can both mimic existing experimental observations as well as suggesting new experimental options for studying collective cell spreading [7]. Such mathematical models have provided key insights into several biological systems. For example, Greenspan's model [8] of tumor growth provided a potential explanation of the observed spatial structure in tumor spheroids, while Gatenby and Gawlinski's model of tumor spreading into surrounding tissue [9] predicted the formation of a gap between the two types of tissue that was later verified experimentally [7].

Almost all mathematical models of collective cell spreading processes make the simplifying assumption that the population of cells can be treated as a uniform population. For example, Maini and coworkers [2], [3] studied a scratch assay and showed that the solution of a reaction–diffusion partial differential equation led to constant-speed, constant-shape moving fronts that were consistent with experimental measurements. Similarly, Sengers and coworkers [10], [11] studied a circular cell spreading assay and showed that the solutions of an axisymmetric reaction–diffusion equation matched the time evolution of the observed experimental cell density profiles. These studies made an implicit assumption that the motion of cells within the population could be described using a constant value of the cell diffusivity 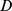, and that the proliferation rate of cells could be described by a constant value of the cell proliferation rate, 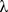. Similar assumptions are often made in discrete models of collective cell motion [12]. For example, Cai and coworkers [13] used a random walk model to study experimental observations of a scratch assay where the motility of isolated individual agents and the birth rate of isolated individual agents in the discrete models were treated as constants. Similarly, Binder and coworkers [14] applied a discrete random walk model of cell migration and cell proliferation on a growing tissue while Khain and coworkers [15] applied a discrete random walk model incorporating cell migration, cell proliferation and cell-to-cell adhesion to a scratch assay performed with glioma cells. Khain's discrete model treated the cell motility, cell proliferation rate and cell-to-cell adhesion strength as a constant for each isolated agent in the simulations.

In contrast to many mathematical models, there are a range of experimental observations which suggest that cell motility and cell proliferation rates are not constant and might vary considerably amongst a population of cells. For example, during the development of the drosophila nervous system, time-lapse observation of individual glia cell migration and proliferation have reported the formation of glial chains which appear to be an essential component of normal development [16], [17]. Time-lapse imaging and cell ablation experiments suggest that a certain subpopulation of the glial cells act as *pioneer* (or *leader*) cells, and that these pioneer cells guide the behavior of the remaining *follower* cells. A similar chain migration model has been proposed to explain time-lapse observations of the development of the enteric nervous system which involves a population of precursor cells, called neural crest cells, moving along the developing intestines in the form of chains of cells [18]–[22]. The details of this developmental system have been studied experimentally and the results suggest that cells at the leading edge of the population follow directed trajectories whereas cells located behind the leading edge of the population followed less directed, more random trajectories [19]. These observations have been recently incorporated into a discrete mathematical model of observed behavior in a related experimental system [23], [24] where it was found necessary to make an explicit distinction between the behavior of pioneer and follower cells to replicate the observed patterns [25].

Experimental observations that are consistent with the existence of pioneer and follower cells have also been made in various *in vitro* assays. For example, Cai and coworkers recorded trajectories of individual cells within a scratch assay and showed that cells at the leading edge of the population moved along trajectories that were qualitatively different to other cells located behind the leading edge [13]. Distinct roles for pioneer and follower cells have been observed in cell populations that interact with collagen fibres [26] and in two–dimensional monolayers of cells that have been wounded [27]. Other biological systems which suggest a role for pioneer and follower subpopulations of cells include the immune system [28]–[30], three—dimensional tumor spheroid growth [31] and various aspects of development [32], [33]. We note that, very recently, heterogeneity amongst circulating tumor cells in patients with advanced primary cancer has been proposed to explain variations in metastatic disease patterns [34].

In this work we investigate whether an apparently homogeneous population of motile cells is composed of functionally distinct subpopulations that could be interpreted as a pioneer subpopulation and a follower subpopulation. This investigation makes use of both experimental measurements as well as a simplified mathematical model of collective cell behaviour that we use to represent both individual cell behavior and the emergent collective behavior of the entire cell population. We perform a three–dimensional transwell assay [35] where we stop the experiment after a relatively short period of time and remove those cells which have moved through the porous membrane as well as those cells which have not moved through the porous membrane. Both these populations of cells are cultured separately, and individual cell trajectories are recorded so that we can investigate whether there are any differences between the two groups of cells. Our experimental measurements are interpreted using a discrete three-dimensional mathematical model of cell migration in a transwell. Although, in principle, our mathematical model can be used to study a very general population of cells where each cell has a unique motility and proliferation rate, we take the simplest possible approach and interpret our experiments by making the assumption that the total population is composed of just two subpopulations which we refer to as (i) subpopulation 1 with cell diffusivity, 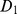, and cell proliferation rate, 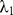, and (ii) subpopulation 2 with cell diffusivity, 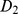, and cell proliferation rate, 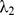. Using our model we show that our transwell results are consistent with the hypothesis that the two subpopulations are distinct since we find 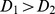. Although we make no experimental measurements of collective behavior involving cell proliferation, we conclude by presenting some simulations of a scratch assay where proliferation plays an important role. In these simulations we treat the entire population as two interacting subpopulations and our modeling suggests that an initially well–mixed population of cells can form a spatially organized spreading front of cells where the more motile subpopulation dominate at the leading edge of the spreading population whereas the less motile subpopulation is practically absent from the leading edge.

## Materials and Methods

### 1.1 Experimental methods

Mouse fibroblast feeder cells [36] (3T3 cells) (ATCC, CCL-92, Manassas, VA, USA) were used to perform the transwell migration assay. The 3T3 cells were cultured in Dulbeccos modified Eagle medium (DMEM; Invitrogen, Australia) supplemented with 5% foetal calf serum (FCS; Hyclone, New Zealand), 2 mM L-glutamine (Invitrogen) and 1% v/v Penicillin/Streptomycin (Invitrogen) in 5% CO_2_ and 95% air at 37°C.

**Figure 1:**
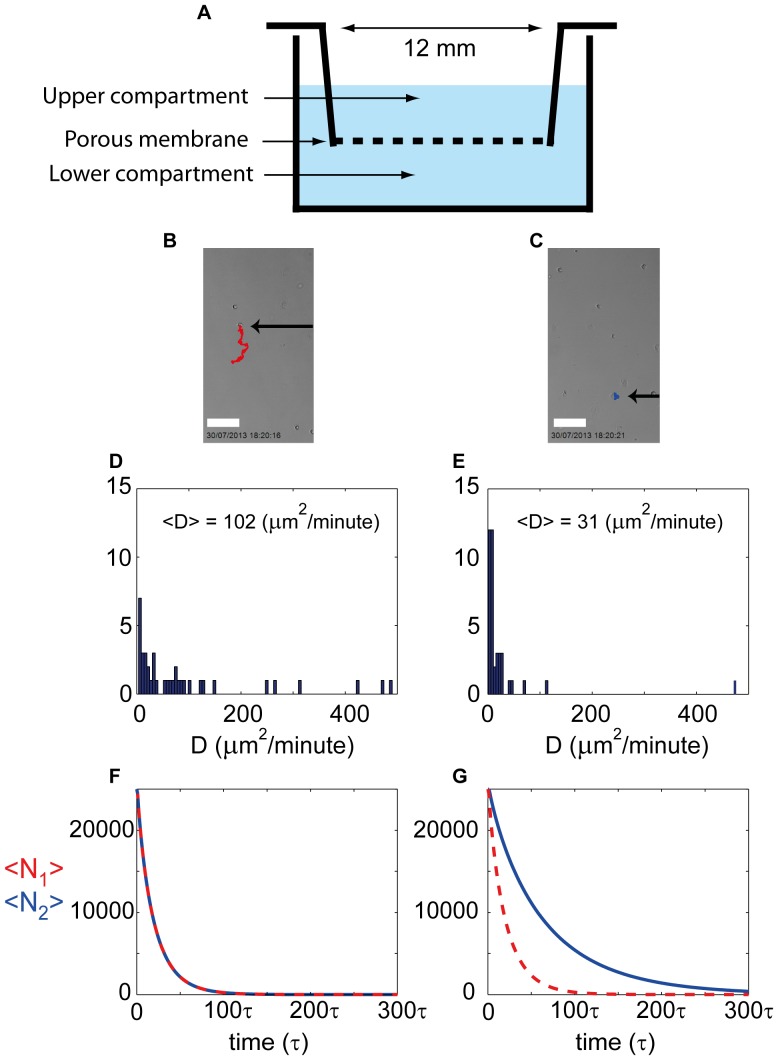
Experimental results and three dimensional mathematical modeling results for a transwell assay. Schematic of a transwell apparatus illustrating that the cylindrical insert is 12(A). At the conclusion of the two hour transwell migration assay those cells that moved into the lower compartment were collected and placed on a cell culture plate. The trajectories of individual cells were recorded over a period of 16 hours. The white scale bar is 100 µm (B). Similarly, at the conclusion of the two hour transwell assay those cells that remained in the upper compartment of were collected and placed on a tissue culture plate. The trajectories of individual cells were recorded over a period of 16 hours. The white scale bar is 100 µm (C). The trajectories of 20 individual cells from those that moved into the lower compartment were analyzed to produce 40 estimates of the cell diffusivity 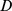, shown as a histogram (D). The average cell diffusivity of those cells that had moved into the lower compartment was 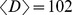 µm^2^/minute. The trajectories of 20 individual cells from those cells that remained in the upper compartment of the transwell were analyzed to produce 40 estimates of the cell diffusivity 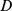, shown as a histogram (E). The average cell diffusivity of those cells that had not moved into the lower compartment of the transwell was 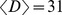 µm^2^/minute. Three dimensional simulation results of a transwell assay initialized with 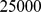 cells from subpopulation 1 and 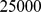 cells from subpopulation 2 (F–G). Simulation results show 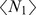 and 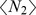, corresponding to the average number of cells associated with subpopulation 1 and subpopulation 2 remaining in the upper compartment as a function of time. The average simulation results were obtained using 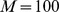 identically prepared realizations of the three dimensional random walk model. Simulation results correspond to two cases: (i) identical subpopulations with 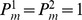 and 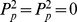 (F), and (ii) distinct subpopulations with 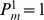, 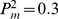 and 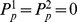.

A schematic of the transwell apparatus is shown in Fig. 1A, and the assay was performed as previously described [37]. In brief, the 3T3 cells were serum starved for four hours by incubating in serum free medium (SFM). The SFM was DMEM without FCS. The cells were harvested, and the flasks washed with phosphate–buffered saline (PBS; Invitrogen) followed by exposure to 0.05% trypsin–EDTA (Invitrogen) for one-to-two minutes at room temperature. The cell suspension was collected in a 50 mL falcon tube and centrifuged twice at 1000 rpm for five minutes to eliminate the trypsin. The supernatant was discarded and the pellet re-suspended in 10 mL of SFM. The viable cells were counted using a trypan blue exclusion test and a haemocytometer.

Fifty thousand 3T3 cells suspended in SFM were seeded into the upper compartment of a 12 µm pore transwell (Corning, New York, USA) where the under-surface of the porous membrane had been pre-coated with with 10% FCS. Each transwell was placed in a 12—well plate which was incubated for two hours at 37°C with 5% CO_2_ and 95% air. After two hours, those cells that had moved into the lower compartment and those cells that remained in the upper compartment were collected separately using 0.05% trypsin–EDTA. The transwell inserts were first rinsed with PBS and then trypsin was introduced into the upper and lower compartments to collect the two groups of cells separately. The collected cells were centrifuged at 1000 rpm for five minutes to remove trypsin and re-suspended in 1 mL of 3T3 medium.

Both groups of cells were separately re-seeded onto a 24–well tissue culture plate and monitored using a widefield microscope (Leica, Australia). Images were captured at five minute intervals over a period of 16 hours.

### 1.2 Mathematical modeling tools

An interacting random walk model, that explicitly incorporates cell-to-cell crowding effects, is used to simulate the experiments. The model is implemented on a three—dimensional square lattice with spacing 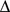. Each site is indexed 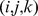, where 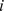, 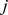, 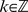, and has position 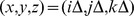. A random sequential update method [38] is used to perform the simulations so that if there are 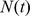 agents at time 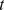, during the next time step of duration 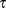, 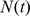 agents are selected at random, one at a time, and given the opportunity move with probability 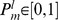, where 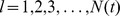. Specifying different values of 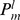 allows different agents in the model to move with a different, unique, motility rate. A motile agent at site 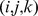 attempts to step to: (i) 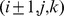 with probability 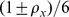, (ii) 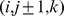 with probability 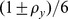, or (iii) 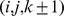 with probability 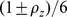. The parameters 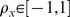, 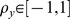 and 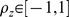 control the motility bias: setting 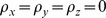 means that the motion is unbiased. If an agent attempts to step to an occupied site, then that motility event is aborted. Once the 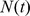 potential motility events have been assessed, another 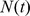 agents are selected at random, one at a time, and given the opportunity to proliferate with probability 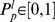. We model proliferation with an unbiased mechanism whereby a proliferative agent at 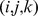 attempts to deposit a daughter agent at 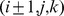, 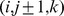 or 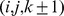, with each target site chosen with equal probability 1/6. Potential proliferation events that would place an agent on an occupied site are aborted [35], [39], [40].

This basic modeling framework will be applied to two different experimental scenarios. First, we will apply this three–dimensional model directly to the geometry of the transwell apparatus as we have done previously [35]. Second, we will consider a simpler two–dimensional application of the model which is consistent with a two–dimensional *in vitro* assay, such as a scratch assay [41]. We note that the three–dimensional modeling framework can be used to simulate a two–dimensional assay simply by considering a three–dimensional lattice with a single layer in the vertical direction, so that 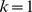. In the simpler two–dimensional format a motile agent at site 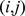 will attempt to step to: (i) 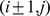 with probability 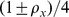, or (ii) 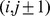 with probability 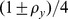. Similarly, a proliferative agent at 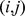 attempts to deposit a daughter agent at 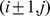 or 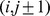, with each target site chosen with equal probability 1/4.

Although, in principle, our discrete modeling framework can be applied to a very general system by allowing every single agent within the population to have a unique motility and proliferation rate, we will implement our model using the simplest possible way to investigate the role of variability within the total population by making the assumption that the population is composed of two subpopulations: (i) subpopulation 1, which is composed of cells which have a probability of motility per time step of 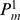 and a probability of proliferation per time step of 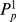, and (ii) subpopulation 2, which is composed of cells which have a probability of motility per time step of 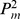 and a probability of proliferation per time step of 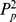 [40].

We would like to point out that while our mathematical model explicitly incorporates physical interactions between cells in the population by incorporating cell–to–cell crowding and volume exclusion effects, our mathematical model is an idealization of collective cell behaviour. One important aspect that our model neglects is any consideration of biochemical interactions amongst the population of cells, which can play an important role in collective cell behaviour [20], [21]. The neglect of such biochemical interactions is a standard assumption made in many mathematical modelling studies of collective cell migration [2]–[5], [8], [10], [11], [13], [15] and the focus of our present work is not to build a mathematical model which incorporates every detail of collective cell migration. Instead, the focus of our present work is to investigate the role of variability amongst a population of cells since traditional mathematical models of collective cell behaviour routinely treat the motility of cells as a simple constant value across a population of cells [2], [3], [10], [11], [13]. Similarly, most traditional mathematical models of collective cell behaviour routinely treat the proliferation rate of cells as a simple constant value across a population of cells [2], [3], [10], [11], [13]. The aim of our work is to explore the validity of such assumptions and to use a simplified mathematical model to demonstrate the implications of such assumptions.

## Results

### 2.1 Transwell Results

#### 2.1.1 Estimating the cell diffusivity

Once the cells were harvested at the conclusion of the two hour migration period in the transwell apparatus, those cells that had migrated into the lower compartment of the transwell (Fig. 1A) were collected separately from those cells that remained in the upper compartment (Fig. 1A). These two groups of cells were placed on separate culture plates and individual cells within the two groups were imaged using time-lapse microscopy for a period of 16 hours so that we could characterize the motility of both populations. At the conclusion of the 16 hour period the time-lapse images were analyzed using ImageJ to record the trajectories of individual cells within the population [42]. For simplicity we will refer to those cells that migrated through into the lower compartment of the transwell as subpopulation 1, and those cells that remained in the upper compartment of the transwell as subpopulation 2.

To characterize the motility we estimate the squared displacement for the 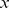-coordinate and 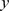-coordinate of each trajectory
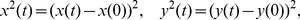
(1)where 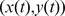 are the two–dimensional Cartesian coordinates of the cell after time 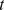. An estimate of the random motility coefficient, also known as the cell diffusivity, in each orthogonal direction is then obtained by fitting a least–squares straight line to the data [43],
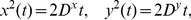
(2)where 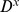 and 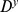 are the diffusivities in the 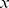 and 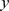 directions. We analyzed 20 randomly chosen cell trajectories from each subpopulation, being careful that we only considered trajectories that did not collide with other cells during the 16 hour observation period. This gave us 20 estimates of 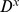 and 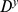 for both subpopulations. Averaging these data, for both subpopulations, indicated that 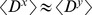 which is reasonable since the substrate is isotropic [44]. Therefore, for each subpopulation we pooled the 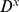 and 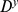 data which are presented as histograms in Fig. 1D and Fig. 1E for subpopulations 1 and 2, respectively. In both histograms the data shows that the majority of observed trajectories are associated with a diffusivity in the range 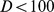 µm^2^/minute. However, both subpopulations contained some cells that were much more motile, and we observed some trajectories corresponding to cell diffusivity estimates with 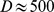 µm^2^/minute. Averaging the 40 diffusivity estimates for each subpopulation gives 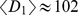 µm^2^/minute and 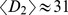 µm^2^/minute. These results indicate that subpopulation 1 is, on average, approximately 3.3 times more motile than subpopulation 2.

#### 2.1.2 Discrete simulations using the transwell data

To investigate how the variations within the cell population could affect our interpretation of a transwell assay we apply the three–dimensional mathematical model to the transwell apparatus using the same procedure outlined previously in [35]. In brief, the transwell is cylindrical with an inner diameter of 12 mm and the 3T3 cells are, on average, approximately 25 µm in diameter [45]. We represent the upper compartment using a three dimensional lattice with 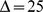 µm. The three dimensional lattice has five layers in the vertical direction giving 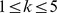, and each layer is a square with length 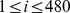 and width 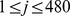. The length and width are chosen to accommodate the 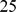 µm 3T3 cells in the 12 mm diameter transwell so that we have 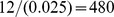. To represent the cylindrical geometry, all sites in the region 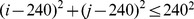 are labeled as *active* sites, meaning that they can can be occupied by agents. The remaining sites where 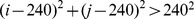 are labeled *inactive* sites, which cannot be occupied by agents. Each layer in the lattice contains 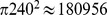 active sites so that our model can accommodate up to 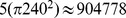 agents. The porous membrane separates the upper and lower compartments and is approximately 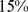 pore space [35]. To model the porous membrane we randomly select 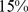 of the active sites on the lower 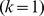 layer of the lattice and assume that these sites, called *downward permeable sites*, represent a pore in the membrane. The remaining 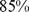 of active sites on the lower 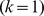 layer are *downward impermeable sites*. In our model a motile agent residing on a *downward impermeable site* 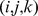, steps to (i) 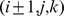 with probability 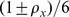, (ii) 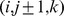 with probability 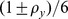, and (iii) 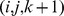 with probability 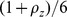 and 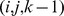 with probability zero owing to the presence of the porous membrane. In comparison, a motile agent residing on a *downward permeable site* 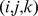 is permitted to move in the negative 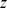 direction in the usual way as this agent is not blocked by the membrane.

During a transwell assay cells are placed in the upper compartment and rapidly settle onto the porous membrane [35]. We model this by placing agents on the lattice to mimic the way that cells are distributed after they have settled onto the membrane. For example, to model our experiments described in Section 1.1 we initially randomly occupy 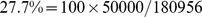 of active lattice sites on the lower 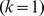 layer of the lattice. To represent the movement of cells in the transwell experiments we set 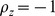 to prevent agents moving vertically upward which is consistent with our observations of cell movement in a transwell [35]. We also set 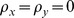, which is appropriate because we do not expect any bias in the horizonal plane. During the simulations some agents move vertically down through the pore space and we assume that these agents leave the system and no longer interact with other agents during that simulation. Any potential motility event that would place an agent on an inactive site, or on a site that is already occupied, is aborted [35], [39], [40]. Our model predictions are made by counting the number of agents leaving the system through the lower layer of the lattice. Since the algorithm is stochastic we present results by averaging over many identically prepared realizations of each simulation.

Results in Fig. 1F correspond to a simulation where the transwell experiment was initialized with 25000 agents from subpopulation 1 and 25000 agents from subpopulation 2. In this case we make the standard assumption that both subpopulations are identical with 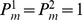. We note that many transwell assays are performed for periods of time that are much shorter than the cell cycle time [35]. This means that any increase in cell number due to cell proliferation is negligible during such experiments. To make our modeling consistent with this we set 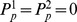. Averaged modeling results in Fig. 1F show the number of agents in each subpopulation that remain in the upper compartment as a function of time and we see that the time taken for both subpopulations to exit the upper compartment are the same. After approximately 100 time steps almost all of the agents have moved into the lower compartment. This result makes sense intuitively since we have specified that both subpopulations behave identically so we might have anticipated that both subpopulations will exit the upper compartment of the transwell at the same rate. We would like to point out that the results in Fig. 1F are reported for an arbitrary duration of each time step, 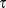. If, for example, we chose 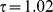 minutes, our simulations would correspond to 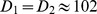 µm^2^/minute since we have 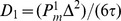 and 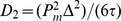.

Results in Fig. 1G correspond to a simulation where the transwell experiment was initialized with 25000 agents from subpopulation 1 and 25000 agents from subpopulation 2. In this case we assume that the subpopulations are distinct and we choose the motility parameters to reflect the differences we observed in the experimental data reported in Section 2.1.1. By choosing 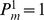 and 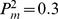, we simulate two distinct subpopulations where subpopulation 1 is approximately 3.3 times more motile than subpopulation 2. Again, to be consistent with standard transwell protocols, we neglect any increase in cell number by cell proliferation by setting 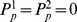 [35]. The averaged modeling results in Fig. 1G show that we observe very different behavior from the results in Fig. 1F where we made the standard assumption that all the cells agents in the system behaved identically. In this case our modeling shows that subpopulation 1 moves into the lower compartment much faster than subpopulation 2 (Fig. 1G). In particular, we see that after 100 time steps almost all of subpopulation 1 has moved into the lower compartment whereas almost 300 time steps are required for almost all of subpopulation 2 to move into the lower compartment (Fig. 1G). This difference in the behavior of the two subpopulations is expected since we have 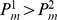, and so we anticipate that agents from subpopulation 1 are able to migrate around in the transwell much more efficiently than members of subpopulation 2. This would mean that agents belonging to subpopulation 1 are more likely to find the location of the pores in the membrane through which they can move into the lower compartment. We also note that the results in Fig. 1G are reported for an arbitrary duration of each time step 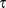. If, for example, we chose 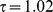 minutes, then this would correspond to 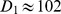 µm^2^/minute and 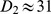 µm^2^/minute which is consistent with our cell diffusivity estimates from our experiments as reported in Fig. 1D and Fig. 1E.

In summary, our modeling results indicate that our interpretation of transwell assays could be very sensitive to differences amongst the motility rates of the cells. Examining the results in Fig. 1G indicates that if we stopped the simulation after a relatively short period of time, say 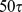, then almost all of subpopulation 1 would have moved into the lower compartment while the majority of subpopulation 2 would remain in the upper compartment. These averaged simulation results are consistent with our experimental observations in Fig. 1D and Fig. 1E since our experimental data indicates that the group of cells that moved into the lower compartment after a relatively short time period were, on average, more motile than the group of cells remaining in the upper compartment.

### 2.2 Scratch assay

Since our modeling results in Fig. 1F and Fig. 1G imply that a transwell assay could be very sensitive to differences amongst the motility rate of the cell population, we now extend these ideas to a scratch assay [2], [3], [41]. Scratch assays are often performed in a narrow channel geometry where a confluent population of cells is wounded, or scratched, to reveal a sharp front that separates the confluent region from a vacant region. Typically, a scratch assay is monitored by measuring the location of the leading edge of the population as it spreads and the initially vacant region becomes occupied [2], [3], [41]. To model this we apply the discrete mathematical model on a two–dimensional lattice where each site is indexed 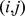, and each site has position 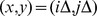. Here we choose 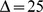 µm to correspond to the diameter of 3T3 cells. We apply this model on a two-dimensional domain with 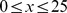 mm and 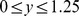 mm, to mimic the narrow channel geometry. Reflecting boundary conditions are applied along all boundaries.

**Figure 2:**
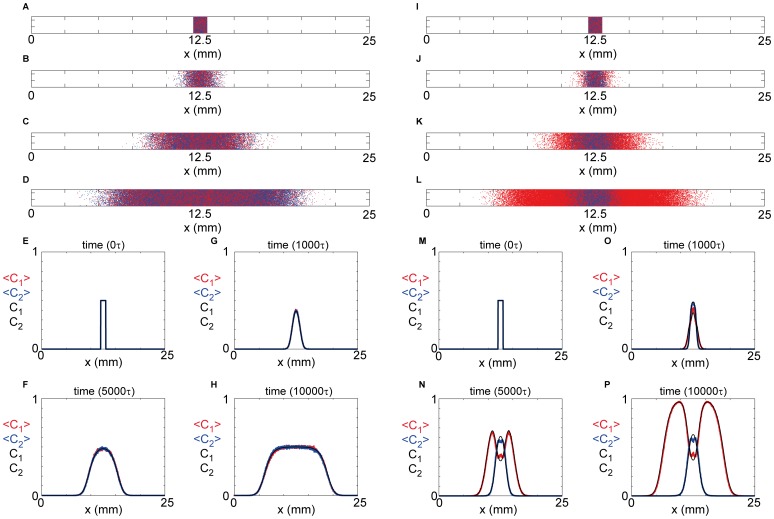
Two-dimensional modeling results for a scratch assay. Discrete snapshots of a two dimensional scratch assay in a narrow channel geometry with 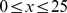 mm and 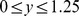 mm (A–D, I–L). The initial condition for two different simulations corresponds to a confluent monolayer of agents in the central region of the domain, where 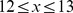 mm. The initial population is made up of 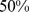 subpopulation 1 (red disks) and 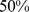 subpopulation 2 (blue disks). The first simulation corresponds to identical subpopulations with 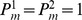 and 
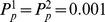
 (A–D) and the second simulation corresponds to distinct subpopulations with 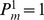, 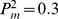, 
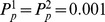
 (I–L). Snapshots are shown after 0 (A,I), 1000 (B,J), 5000 (C,K) and 10000 (D,L) time steps, where each time step has a duration of 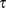. Both types of discrete simulation were repeated using 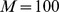 identically prepared realizations to to produce the averaged density profiles for the case where both subpopulation are identical (E–H) and where the subpopulations are distinct (M–P). The numerical solution of Equation (5) was obtained for the initial condition given by Equations (8)–(9) and superimposed on the averaged discrete results (E–H, M–P). The numerical solutions were obtained using 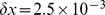 mm and 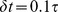.

To be consistent with our results in Fig. 1F and Fig. 1G, we consider the initial population of agents to be composed of two subpopulations. Each simulation is initialized so that the central region of the lattice, where 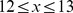 mm, contains a confluent monolayer. This initial confluent monolayer contains, on average, 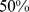 of agents from subpopulation 1 and 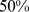 of agents from subpopulation 2. Two different types of simulations are performed. In the first simulation (Fig. 2A–D) we make the standard assumption that both subpopulations are identical with 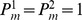 and 
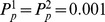
. Unlike transwell assays, many scratch assays are reported for a period of time that is longer than the cell cycle time so that proliferation plays an important role [2], [3], [41] and therefore we include proliferation in these simulations [39]. Results in Fig. 2A–D show snapshots of the simulation after 0, 1000, 5000 and 10000 time steps, where each time step has a duration 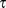. These simulations show that the population spreads into the initially vacant region. Individual agent motility and proliferation events lead to the formation of two fronts, one moving in the positive 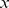
–direction and the other moving in the negative 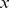
–direction. The formation of such fronts is consistent with experimental observations where these fronts often move with constant speed [2], [3]. We observe that the total population grows rapidly with time, and our simulation indicates that the two subpopulations remain well–mixed for all time and at all locations. The results in Fig. 2A–D are reported for an arbitrary duration of each time step, 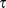. If, for example, we chose 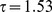 minutes, this would correspond to 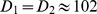 µm^2^/minute since we have 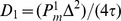 and 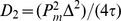 in two–dimensions. Similarly, choosing 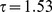 minutes corresponds to 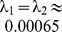/minute since we have 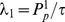 and 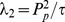. This proliferation rate corresponds to a doubling time of 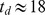 hours since we have 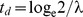 [35].

In the second simulation (Fig. 2I–L) we allow the two subpopulations to behave differently by setting 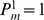 and 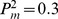, so that subpopulation 1 is approximately 3.3 times more motile than subpopulation 2. Again, this difference in the motility rate between the two subpopulations is consistent with our experimental results in Fig. 1D and Fig. 1E. Since we have not made any measurements of the proliferation rate of cells we assume that both subpopulations proliferate at the same rate with 
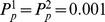
. Results in Fig. 2I–L show a snapshot of the simulation after 0, 1000, 5000 and 10000 time steps, where each time step has a duration of 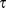. Just like the uniform population in Fig. 2A–D, we see that the population spreads into the initially vacant region of the domain and the model predicts the formation of two fronts, one moving in the positive 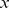
–direction and the other moving in the negative 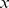
–direction. Again, the total population grows rapidly with time, however in this case our results indicate that the two initially well–mixed subpopulations remain mixed for a short period of time only (Fig. 2I–J) before becoming segregated at later times (Fig. 2K–L) where we see that the leading edge of the population is dominated by subpopulation 1. This result implies that the leading edge of the spreading population becomes dominated by the subpopulation that is more motile. The results in Fig. 2I–J are reported for an arbitrary duration of each time step, 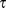. If, for example, we chose 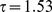 minutes, this would correspond to 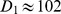 µm^2^/minute and 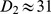 µm^2^/minute which is consistent with our experimental observations in Fig. 1D–E.

### 2.3 Continuum description

The simulation results in Fig. 2A–D and Fig. 2I–L correspond to single realizations of the discrete model. To provide additional information about these simulations we consider 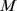 identically prepared realizations and generate averaged density profiles. In the 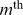 identically prepared realization of the model, site 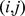 can be either, (i) occupied by an agent from subpopulation 1, 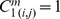, (ii) occupied by an agent from subpopulation 2, 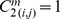, or (iii) vacant with 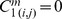 and 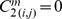. From our simulations we can estimate the average occupancy of agents from subpopulation 1 at site 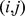 as 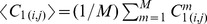, and the average occupancy of agents from subpopulation 2 at site 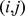 as 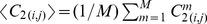.

Results in Fig. 2E–H show 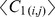 and 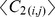 associated with the simulations in Fig. 2A–D for 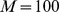. These averaged profiles confirm that both subpopulations spread across the domain with time and form two moving fronts, one moving in the positive 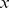
– direction and the other moving in the negative 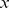
–direction. The averaged density profiles in Fig. 2E–H confirm that both subpopulations remain well mixed since we have 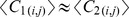 at all locations and for all time. Results in Fig. 2M–P show 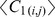 and 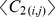 associated with the simulations in Fig. 2I–L for 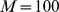. These profiles confirm that two moving fronts of cells form with time and that one moves in the positive 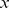 –direction and the other moving in the negative 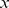
–direction. The averaged density profiles in Fig. 2M–P show that the two subpopulations do not remain well mixed since we see that the leading edge of the moving fronts are eventually dominated by subpopulation 1.

To describe these averaged simulation results using a continuum mathematical framework we form two discrete conservation statements for 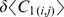 and 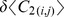, which describe the the change in average occupancy of subpopulation 1 and 2, respectively, at site 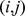, during the time interval from time 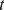 until time 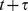. The discrete conservation statements are given by
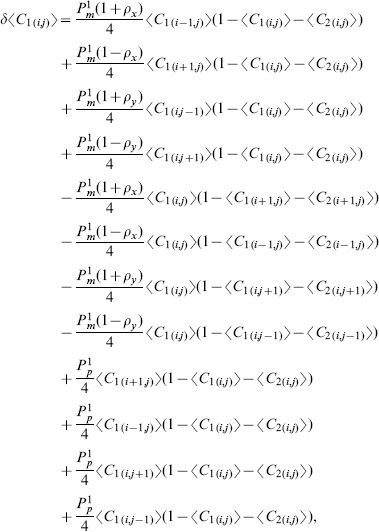
(3)and
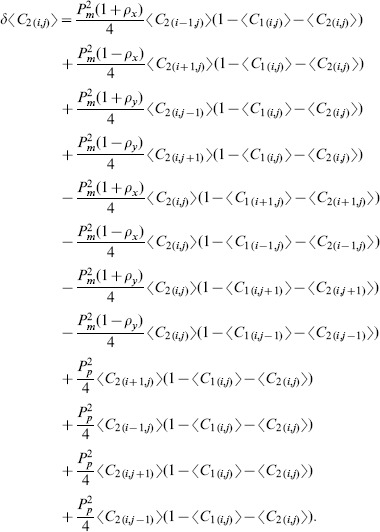
(4)Positive terms on the right of Equation (3) represent events that place an agent of subpopulation 1 at site 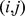, while the negative terms on the right of Equation (3) represent events that remove agents of subpopulation 1 from site 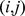. A equivalent interpretation applies to the terms on the right of Equation (4) with respect to agents from subpopulation 2. All the terms on the right of Equations (3) and (4) involve factors like 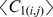 and 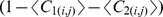 which we interpret the probability that site 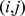 is occupied by an agent from subpopulation 1, or the probability that site 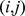 is vacant, respectively. We interpret products of these terms as net transition probabilities which means that we are making the usual assumption that the occupancy of lattice sites are independent [46]–[48]. As we shall later demonstrate (Fig. 2) this assumption appears to be a reasonable for the problems we consider here.

The discrete conservation statements, given by Equation (3) and (4) are related to a system of partial differential equations in the appropriate limit as 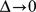 and 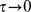 and the averaged data, 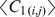 and 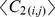 are written in terms of two continuous variables 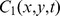 and 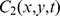. To find this relationship we expand all terms in Equations (3) and (4) in a truncated Taylor series about site 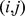, keeping terms up to 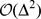. Dividing the resulting expressions by 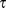, we consider the limit as 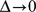 and 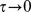 simultaneously, with the ratio 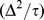 held constant. In the continuum limit, the partial differential equations governing 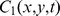 and 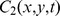 can be written as

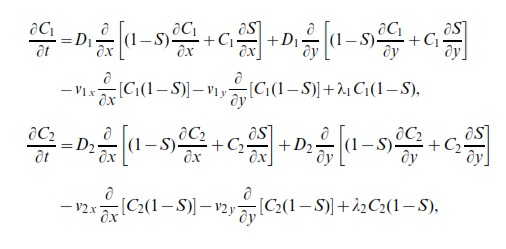
(5)
where

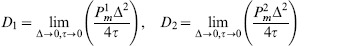


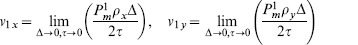


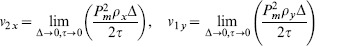


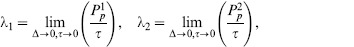
(6)and 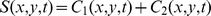 is the total density [35], [39]

We note that for the special case where the motion is unbiased 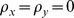, and that both subpopulations are identical with 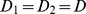, 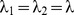, we can re-write Equation (5) in terms of the total population density as
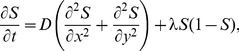
(7)which is the two-dimensional analogue of the well known Fisher Kolmogorov equation [49], [50]. This standard reaction diffusion model is a particular case of the more general system derived here.

We note that Equation (5) is written in terms of the two-dimensional 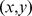 Cartesian plane. If we consider an initial condition, 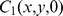 and 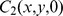, that is independent of the vertical coordinate 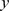, and either periodic or reflecting boundary conditions are applied on both boundaries parallel to the 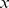 coordinate, the solution of Equation (5) is independent of 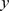 for all 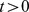 and we have 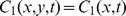 and 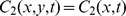 [35], [39], [40]. These initial conditions and boundary conditions are relevant when considering an *in vitro* experiment in a narrow channel geometry, such as a scratch assay [2], [3], [15] or the discrete simulations in Fig. 2A–D and Fig. 2I–L. For other types of assays where the geometry is genuinely two-dimensional, such as barrier assays [45], [51]–[54], we must consider the complete two-dimensional partial differential equations as demonstrated previously in [40].

To investigate how the solution of Equation (5) relates to the averaged discrete data in Fig. 2E–H and Fig. 2M–P, we solved Equation (5) numerically on 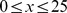 mm with reflecting boundary conditions for both subpopulations at both boundaries. To match the averaged discrete simulation data we use the same initial condition as in the discrete simulations
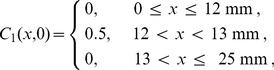
(8)and
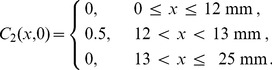
(9)We solve Equations (5) using a non-iterative operator split method [55]. To solve the transport terms in Equation (5) we use central difference approximation with mesh spacing 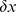, and implicit Euler stepping with a time step of 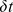. To solve the reaction terms in Equation (5) we use a fourth order Runge-Kutta method with time step 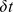 [56].

The numerical solution of Equation (5), with 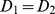 and 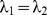, is superimposed in Fig. 2E–H where we see that 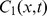 and 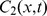 match the averaged discrete data, 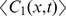 and 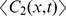, very well at all locations and for all times considered. Similarly, the numerical solution of Equation (5) with 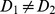 and 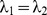 are superimposed in Fig. 2M–P where we also see that these solutions match the averaged discrete data very well at all locations for all times considered. We would like to reiterate here that the key result is that when the subpopulations are identical we have 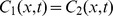 whereas when we consider distinct subpopulation with 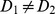, we observe an influence on the spatial and temporal organization of the two subpopulations. In particular, we see that the more motile subpopulation, 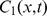, dominates the total population at the leading edge whereas the less motile subpopulation, 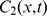, is absent from the leading edge. Therefore, locally at the invasive front we have 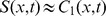 and 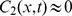. We note that these kinds of differences, where cells at the leading edge of an invasive population are appear to be functionally distinct from cells located well behind the leading edge of the invasive population have also been observed experimentally in various *in vivo* [19] and *in vitro* contexts [13].

## Discussion and Conclusions

Mathematical and computational modeling has played an important role in improving our understanding of collective cell spreading in a range of applications [2]–[5], [13], [22]. Despite a range experimental evidence that suggests otherwise, most mathematical models of collective cell behavior make the simplifying assumption that the cell motility rate and the cell proliferation rate are constants and do not vary amongst the cell population. These kinds of simplifying assumptions give rise to mathematical models that take the form of reaction diffusion equations with constant cell diffusivity [2]–[5], or discrete random walk models of collective cell behavior where isolated individual agents in the system have constant rates of motility [13], [15], [35], [39].

In this work we have sought to explore the validity of these standard assumptions by performing a transwell assay with 3T3 cells. By stopping the assay after a short period of time we aimed to test the hypothesis that those cells amongst the total population with high motility rate would move into the lower compartment of the transwell faster than those cells amongst the total population with a lower motility rate. Indeed our time lapse data suggests that those cells that moved into the lower compartment in a short period of time were, on average, approximately three times more motile that those cells remaining in the upper compartment of the transwell. We illustrated the role that such variability can have by applying an existing model of cell migration through a transwell [35] which we generalize so that each agent in the simulation can have a distinct motility rate and distinct proliferation rate. Taking the simplest possible approach where we consider the total population to be composed of two subpopulations, we show that the mathematical model predicts very different behavior in the transwell assay where we account for differences in the motility rate between subpopulation 1 and subpopulation 2.

We also apply our mathematical model to the situations where we idealize a total population of cells as two possibly distinct subpopulations, to a scratch assay. Our simulations and analysis indicate that when we make the standard assumption that both subpopulations have identical cell diffusivity (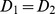) and identical cell proliferation rate (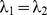), with the further assumption that the initial condition is a well mixed population where both subpopulations are present in equal proportions, we observe the formation of moving fronts of cells where both subpopulations are well mixed throughout. In contrast, if we assume that the subpopulations have distinct cell diffusivities (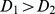) and identical cell proliferation rates (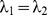) our modeling shows that the moving fronts of cells that forms is very different. In this case the two subpopulations do not remain well mixed, and instead we observe that the subpopulation with the higher diffusivity dominates at the leading edge of the population. This idea that the cells at the leading edge of invasive fronts are more motile than their counterparts well behind the leading edge is consistent with previous experimental observations [13], [19].

There are several ways that the modeling results can be extended. For example, when we considered the scratch assay simulations in Fig. 2 we always made the simplifying assumption that both subpopulations were initially present in equal proportions so that we had 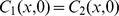. In the case that we have distinct subpopulations with 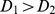, we note that our main result, showing that the two subpopulations do not remain well mixed after a sufficiently long period of time, also holds when we vary the initial condition. For example, our results in Fig. 2 made the assumption that the central region of the domain was equally composed of both subpopulations, 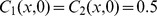 (Fig. 2I). If, instead, we suppose that subpopulation 2 dominates initially by setting 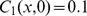 and 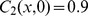 in this central region, our modeling framework predicts that subpopulation 1, with 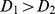, will eventually dominate the leading edge of the spreading front despite the fact that there is only a small proportion of subpopulation 1 present at the beginning of the experiment.

Another simplifying assumption made here is that we supposed that the total population of cells in the system could be idealized as two subpopulations. This assumption was invoked so that we could illustrate our results as simply as possible and we note that our discrete modeling framework, outlined in Section 1.2, and the associated continuum partial differential equation description, can be generalized so that we can consider dividing the total population into an arbitrary number of subpopulations. For example, if instead of treating the total population in Fig. 2 as two subpopulations, we could consider the total population to be composed of 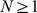 subpopulations. Taking the same approach leads to the following system of coupled partial differential equations
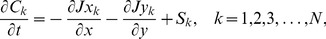
(10)where 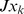 is the flux of subpopulation 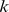 in the 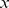
–direction, 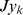 is the flux of subpopulation 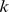 in the 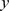
–direction and 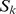 is the source term for subpopulation 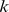. These terms can be written as
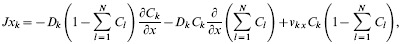


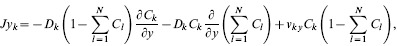


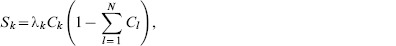
(11)where
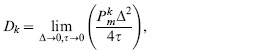


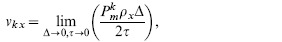


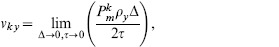


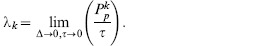
(12)We note that the question of determining the appropriate number of subpopulations, 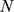, which accurately reflects the collective behavior of a real population of cells is an open question that requires further experimental and theoretical investigation.

Our experimental methods focused on a transwell assay which are typically conducted over an interval of time that is much shorter than the cell cycle time [35]. As a consequence, our experimental methods were aimed at investigating the variability of cell motility amongst the population rather than focussing on the variability of the cell proliferation rate. To make such an assessment, a different kind of experimental system could be considered, such as a barrier assay [45], [51]–[53] or a scratch assay [2], [3], [41], which are often conducted for periods of time that are longer than the doubling time. We leave such an investigation of the role of variations in the proliferation rate of cells for future investigation.

We conclude with a brief discussion about the limitations of our mathematical modelling framework, together with a brief discussion about the suitability of our mathematical modelling framework for this particular study. One of the limitations of our mathematical model is that it neglects to explicitly incorporate any details regarding biochemical cell to cell interactions. It is important to acknowledge this limitation since biochemical cell to cell interactions are thought to influence collective cell behaviour [20], [21]. Since the focus of our work is to explore the role of variability amongst a population of cells, it is appropriate for us to use a mathematical modelling framework that can explicitly examine the role of variability rather than a mathematical model that incorporates, potentially complicated, biochemical cell to cell interactions. Once again, we would like make the point that many traditional mathematical models of collective cell behaviour treat the motility of cells as a simple constant value across a population of cells [2], [3], [10], [11], [13]. Similarly, many traditional mathematical models of collective cell behaviour treat the proliferation rate of cells as a simple constant value across a population of cells [2], [3], [10], [11], [13]. In contrast, our experimental data showed that measurements of cell diffusivity from a single population of cells can lead to a wide range of cell diffusivity estimates and our modelling framework showed that the neglect of this variability leads to significantly different predictions than when this variability is incorporated.
